# Response Surface Methodology Optimization of Exosome-like Nanovesicles Extraction from *Lycium ruthenicum* Murray and Their Inhibitory Effects on Aβ-Induced Apoptosis and Oxidative Stress in HT22 Cells

**DOI:** 10.3390/foods13203328

**Published:** 2024-10-20

**Authors:** Yadan Zhang, Ling Lu, Yuting Li, Huifan Liu, Wenhua Zhou, Lin Zhang

**Affiliations:** 1 Hunan Key Laboratory of Processed Food for Special Medical Purpose, College of Food Science and Engineering, Central South University of Forestry and Technology, Changsha 410004, China; 20210100084@csuft.edu.cn (Y.Z.);; 2 Hunan No. 1 Health Agriculture Development Co., Ltd., Changsha 411499, China; 3 College of Food Science and Technology, Zhongkai University of Agriculture and Engineering, Guangzhou 510225, China

**Keywords:** *Lycium ruthenicum* Murray, response surface methodology, exosome-like nanovesicles, apoptosis, oxidative stress

## Abstract

Exosome-like nanovesicles (ELNs) derived from plants are nanoscale vesicles isolated from edible plant sources. *Lycium ruthenicum* Murray (LRM) has garnered growing attention for its dietary value and therapeutic benefits. In this study, a PEG6000-based method was developed to isolate LRM-ELNs. Response surface methodology (RSM) was used to optimize the extraction conditions to obtain the optimal extraction efficiency. When PEG6000 concentration was at 11.93%, relative centrifugal force was 9720 g, and incubation time was 21.12 h, the maximum LRM-ELN yield was 4.24 g/kg. This optimization process yielded LRM-ELNs with a particle size of 114.1 nm and a surface charge of −6.36 mV. Additionally, LRM-ELNs mitigated Aβ-induced apoptosis in HT22 cells by enhancing mitochondrial membrane potential (MMP), lowering the Bax/Bcl-2 ratio, and reducing Cleaved Caspase-3 expression. Furthermore, LRM-ELNs alleviated Aβ-induced oxidative stress in HT22 cells by promoting the nuclear translocation of Nrf2 and upregulating the expression of HO-1 and NQO1. These findings indicate that LRM-ELNs exert protective effects against Aβ-induced damage in HT22 cells and may be considered as a potential dietary supplement for Alzheimer’s disease prevention.

## 1. Introduction

Nanoscale extracellular vesicles are secreted by eukaryotic cells, which include those derived from mammals, microorganisms, and plants [1]. Among these, exosomes, a subtype of small extracellular vesicles, are critical for intercellular communication, enabling the transport of various biomolecules such as lipids, proteins, DNA, and RNA [2,3]. Recent advancements have seen exosomes from microorganisms and animal cells being extensively utilized in biomedical applications, particularly in diagnostics, therapeutics, and drug delivery [4,5,6]. Exosomes containing miR-15a-5p from plasma have been demonstrated to serve as effective biomarkers for diagnosing endometrial cancer [4]. Exosomes derived from *lactobacilli* have been shown to inhibit HIV-1 infection [5]. Exosomes from human cells have been demonstrated to deliver miR-497, which exerts anti-cancer effects by inhibiting angiogenesis and tumor growth [6]. Despite their potential, significant challenges remain in efficient production and ensuring biosafety [7]. Plant cells also secrete exosome-like nanovesicles (ELNs), offering advantages such as high yield, security, biological compatibility, and minimal negative impact on the intestinal barrier and other organs. ELNs derived from plant have shown substantial biological activity, representing a promising area for therapeutic development [8,9]. The standard approach for isolating plant derived-ELNs is differential ultracentrifugation, which, despite its effectiveness, is cost-prohibitive and not scalable due to the high expense of equipment and consumables, as well as the limited capacity for processing large sample volumes [10]. Additionally, the high centrifugal forces during ultracentrifugation can potentially disrupt ELN integrity [7]. An alternative approach using polyethylene glycol (PEG) for precipitation has recently been proposed for selective isolation of ELNs derived from ginger [10]. This approach removes the requirement for ultracentrifugation, offering a more economical and easily expandable solution while maintaining comparable yield and bioactivity between ginger ELNs extracted via ultracentrifugation and PEG [10]. The PEG-based method has been successfully employed in various studies to extract plant derived-ELNs. For example, PEG-isolated ELNs from blueberry have demonstrated the ability to improve nonalcoholic fatty liver disease by reducing mitochondrial oxidative stress [11]. Similarly, ELNs isolated from aloe using PEG have demonstrated the ability to inhibit chronic inflammation and facilitate wound healing [12]. Although these findings do not directly compare the activity of ELNs derived from differential ultracentrifugation and PEG, these findings highlight the potential of PEG-based extraction methods as a viable and scalable alternative for obtaining plant ELNs.

Plant-derived ELNs are attracting significant attention as a natural source of antioxidants [13]. For example, Actinidia arguta ELNs were internalized by RAW264.7 cells, which reduced oxidative stress by decreasing malondialdehyde (MDA) levels and increasing total superoxide dismutase (T-SOD) activities and glutathione peroxidase (GSH-Px) levels [14]. Ginseng root ELNs protect the skin from UV radiation and oxidative stress by inhibiting activator protein-1 signaling and reducing reactive oxygen species (ROS) production [15]. Additionally, plant-derived ELNs inhibit oxidative stress associated with neurodegenerative diseases. ELNs derived from carrots markedly reduced ROS production and cell apoptosis triggered by 6-OHDA in SHSY5Y cells, suggesting their potential as a novel candidate for the treatment of Parkinson’s disease [16]. Citrus lemon ELNs exhibit strong antioxidant properties and protect against β-amyloid (Aβ)-induced cytotoxicity in SHSY5Y cells [17]. These findings indicate that plant-derived ELNs have considerable promise for treating oxidative stress associated with neurological disorders.

Alzheimer’s disease (AD) represents a significant subset of neurodegenerative disorders, primarily marked by behavioral disturbances, cognitive decline, and memory loss [18]. Studies on AD pathology reveal a strong correlation between Aβ concentrations and the severity of dementia symptoms [19,20]. The amyloid cascade hypothesis posits that the aggregation of misfolded Aβ peptides initiates oxidative stress, compromising neuronal integrity and function, ultimately leading to cell apoptosis [21,22]. Therefore, reducing Aβ-induced oxidative stress and apoptosis in neural cells could be an effective strategy for treating AD.

*Lycium ruthenicum* Murray (LRM) is a longstanding herbal medicine with well-established therapeutic and dietary uses [23]. Previous research indicates that LRM has significant characteristics, including anti-fatigue, anti-aging, antioxidant, anticancer, and memory-enhancing effects [24,25,26]. A study also suggests that LRM can alleviate key pathological features of AD by modulating the nervous system, immune responses, and signaling pathways [27]. Our preliminary research indicates that LRM-derived ELNs (LRM-ELNs) inhibit Aβ-induced apoptosis in PC12 cells via the PI3K/AKT and MAPK signaling pathways [28]. However, the modulatory effect of LRM-ELNs on oxidative stress induced by Aβ has not yet been thoroughly examined.

This study optimized the extraction conditions of LRM-ELNs using response surface methodology (RSM). Additionally, the impacts of LRM-ELNs on oxidative stress and apoptosis induced by Aβ in HT22 cells were investigated. These findings could offer valuable insights into the high efficiency extraction method and physiological roles of LRM-ELNs and contribute to the development of novel plant-derived ELNs.

## 2. Materials and Methods

### 2.1. Chemicals and Reagents

*Lycium ruthenicum* Murray (LRM) is native to Qinghai Province, China. The LRM used in this study was in a dried form. Aβ_1–42_ was acquired from the Qiangyao Biotechnology Co., Ltd. (Shanghai, China). The HT22 cells were purchased from the American Type Culture Collection (Manassas, VA, USA). Dulbecco’s Modified Eagle Media: Nutrient Mixture (DMEM) cell culture medium and fetal bovine serum (FBS), and penicillin/streptomycin were purchased from Gibco Co., Ltd. (Grand Island, NY, USA). PEG6000 was purchased from Macklin Biochemical Technology Co., Ltd. (Shanghai, China). 3-(4,5-dimethylthiazol-2-yl)-2,5-diphenyltetrazolium bromide (MTT) was obtained from Sigma Chemical Co., Ltd. (St. Louis, MO, USA). Hoechst 33258 and 4% paraformaldehyde/general tissue fixative were purchased from Biosharp Biotechnology Co., Ltd. (Hefei, China). RNase was purchased from Solarbio Technology Co., Ltd. (Beijing, China).

### 2.2. Extraction of LRM-ELNs

LRM-ELNs from LRM were extracted according to previously published methods with minor adjustments [10,29]. Briefly, 50 g of LRM was ground and extracted in a 250 mL of distilled water at a solid/liquid ratio of 1:5 (g/mL). The collected mixtures were centrifuged in a sequential manner at 1000× *g* for 10 min, followed by 3000× *g* for 20 min, and finally at 10,000× *g* for 30 min at 4 °C using an Eppendorf centrifuge (5910R, Hamburg, Germany). The supernatant was treated with PEG6000 and incubated at 4 °C, and after centrifugation for 30 min, the precipitate was collected and subjected to freeze-drying, resulting in the LRM-ELNs (Figure S1).

### 2.3. Single-Factor Experiment

Single-factor experiments were conducted to preliminarily determine the effect of PEG6000 concentration relative centrifugal force and incubation time. In the optimization, each condition was varied to five different values as follows: PEG6000 concentration, 6% to 15%; relative centrifugal force, 2000 to 10,000× *g*; and incubation time, 8 to 24 h.

### 2.4. Design of RSM

Based on the preliminary results obtained in Section 2.2, variations in the concentration of PEG6000, relative centrifugal force, and incubation time were manipulated to achieve the maximum yield of LRM-ELNs. The Box–Behnken design (BBD) was employed in this study using Design Expert v.13.0 software to investigate the impact of three independent variables, namely, PEG6000 concentration (A), relative centrifugal force (B), and incubation time (C), on the yield of LRM-ELNs (Y). The complete experimental design consisted of 17 experimental points (Table S1).

### 2.5. Analysis of LRM-ELNs

To examine the morphology of LRM-ELNs at a concentration of 200 μg/mL, freshly prepared LRM-ELNs were subjected to negative staining using 1% uranyl acetate. Their morphology was then analyzed with a transmission electron microscope (TEM) (Zeiss, LEO 906E, Jena, Germany), and photographic records were taken.

Dynamic light scattering was employed to assess the particle size and surface zeta potential of LRM-ELNs at a concentration of 200 μg/mL and a temperature of 25 °C, using a Zetasizer Nano ZS (Malvern, UK).

### 2.6. MTT Assays

HT22 cells are mouse hippocampal neuronal cells commonly used as models for AD studies [30,31,32]. In this study, HT22 cells were cultured and subsequently transferred into a sterile 96-well plate, with a density of about 1 × 10^4^ cells per well. HT22 cells were subjected to three different treatment conditions: treatment with Aβ alone, treatment with LRM-ELNs alone, or a mixture of Aβ and LRM-ELNs, all for a duration of 24 h. Following this, 100 µL of 10% MTT was supplied to each well and allowed to develop for 4 h. The MTT solution was gently discarded, and 100 µL of DMSO was subsequently introduced. Absorbance was measured at 490 nm using an automated microplate spectrophotometer (Spectra Max i3X, Sunnyvale, CA, USA). Cell viability was reported as a percentage compared to the control.

### 2.7. Flow Cytometry Assay

HT22 cells were seeded into a 6-well plate (3 × 10^5^ cells per well) and allowed to adhere for 6 h. The cells were then exposed to Aβ (20 µM) either alone or in combination with different concentrations of LRM-ELNs (0, 50, 100, 200 µg/mL) for 24 h at 37 °C, with each well containing 2 mL of the respective solution. Apoptosis was assessed using the Annexin V-FITC/PI apoptosis detection kit. After washing the cells with PBS and centrifuging three times, they were treated with Annexin V-FITC and PI solution for 5 min. Cell apoptosis was evaluated using a NovoCyte flow cytometer (Agilent Technologies Inc., Palo Alto, CA, USA).

### 2.8. Measurement of Intracellular Reactive Oxygen Species (ROS)

HT22 cells (3 × 10^5^ cells per well) were plated in a 6-well plate and given time to attach for 6 h. The cells were then exposed to Aβ (20 µM) combined with different concentrations of LRM-ELNs (0, 50, 100, 200 µg/mL) for 24 h at 37 °C, with each well containing 2 mL of the respective solution. After treatment, the cells were processed following the instructions included with the DCFH-DA kit. Images were captured using an inverted fluorescence microscope (Nikon, Tokyo, Japan).

### 2.9. Determination of Intracellular Levels of Superoxide Dismutase (SOD), Catalase (CAT), Glutathione Peroxidase (GSH-Px), and Malondialdehyde (MDA)

HT22 cells (3 × 10^5^ cells per well) were seeded in a 6-well plate and incubated for 6 h. They were then treated with Aβ (20 µM) and varying concentrations of LRM-ELNs (0, 50, 100, 200 µg/mL) for 24 h at 37 °C, with each well containing 2 mL of the respective solution. The activities of SOD, CAT, GSH-Px, and levels of MDA were determined according to the instructions provided with the kits from Nanjing Jiancheng Bioengineering Institute (Nanjing, China).

### 2.10. Determination of Mitochondrial Membrane Potential (MMP)

HT22 cells (3 × 10^5^ cells per well) were seeded onto a 6-well plate and incubated for 6 h. They were then treated with Aβ (20 µM) and various concentrations of LRM-ELNs (0, 50, 100, 200 µg/mL) for 24 h at 37 °C, with each well containing 2 mL of the respective solution. Detection was performed using the JC-1 assay kit (Beyotime, Shanghai, China). MMP was determined as the ratio of JC-1 aggregates (red fluorescence) to JC-1 monomers (green fluorescence).

### 2.11. Western Blot (WB) Assessment

Protein extraction was conducted from HT22 cells and measured using a BCA assay kit. The PVDF membrane was blocked with 5% BSA and then treated with primary antibodies overnight at 4 °C. Antibodies for Nrf2 (A0674), HO-1 (A1346), NQO1 (A0047), Bax (A12009), Bcl2 (A19693), and Histone H3 (A17562) were purchased from ABclonal (Wuhan, China). Cleaved Caspase-3 (ARG57512) was obtained from Arigo (Hsinchu City, Taiwan, China). β-actin (66009-1-lg) was procured from Proteintech (Wuhan, China). The membrane was then treated with HRP-conjugated secondary antibodies for 1 h. Protein bands were visualized with enhanced ECL substrate (Abbkine, Wuhan, China).

### 2.12. Statistical Analysis

All data were expressed as mean ± standard error of the mean (SEM) deviation. A two-tailed, unpaired Student’s *t*-test was performed to compare the two groups. One-way ANOVA followed by Tukey’s post hoc test was utilized for comparisons among multiple groups. A *p*-value of less than 0.05 was considered statistically significant. Data analysis and visualization were performed using GraphPad Prism 9.0.

## 3. Results

### 3.1. Optimization of Parameters of LRM-ELN Extraction Using Single-Factor Experiments

#### 3.1.1. Effects of PEG Molecular Weights on Yield and Characteristics of LRM-ELNs

**Figure 1:**
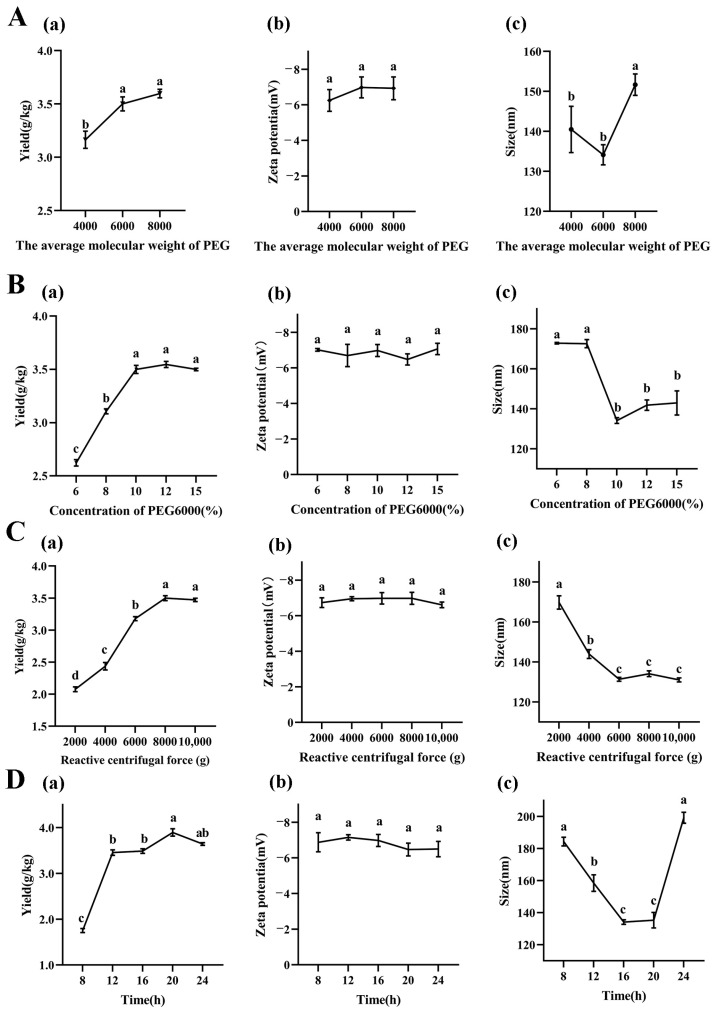
Effect of different extraction conditions on LRM-ELNs. (**A**) The (a) yield, (b) zeta potential, and (c) particle size of LRM-ELNs at different PEG molecular weights. Other extraction conditions: 10% of PEG6000 concentrations, 8000× *g* of relative centrifugal force, and 16 h of incubation time. (**B**) The (a) yield, (b) zeta potential, and (c) particle size of LRM-ELNs at different PEG6000 concentrations. Other extraction conditions: 16 h of incubation time and 8000× *g* of relative centrifugal force. (**C**) The (a) yield, (b) zeta potential, and (c) particle size of LRM-ELNs at different relative centrifugal forces. Other extraction conditions: 10% of PEG6000 concentrations and 16 h of incubation time. (**D**) The (a) yield, (b) zeta potential, and (c) particle size of LRM-ELNs at different incubation times. Other extraction conditions: 10% of PEG6000 concentrations and 8000× *g* of relative centrifugal force. Data are presented as the mean ± SEM. Different letters indicate a significant difference among groups (*p* < 0.05).

To investigate the effects of the molecular weights of PEG on LRM-ELNs characteristics, the yield, zeta potential, and particle size of LRM-ELNs were detected. As shown in Figure 1A(a), with the increase in PEG molecular weights (from 4000 to 6000), the yield of LRM-ELNs was significantly increased, while, when PEG molecular weight increased to 8000, the yield of LRM-ELNs had no significant difference compared to PEG 6000. As shown in Figure 1A(b), the zeta potential of LRM-ELNs produced by different molecular weights of PEG (4000, 6000, 8000) had no significant differences. As shown in Figure 1A(c), PEG6000-isolated LRM-ELNs had the smallest particle size. Nanoparticle size has been shown to affect the transport of particles across different biological barriers, such as the blood–brain barrier (BBB), and smaller particle sizes have higher particle transport [33,34]. Therefore, considering both the yield and size of LRM-ELNs, PEG6000 was selected for further study.

#### 3.1.2. Effects of PEG6000 Concentration on Yield and Characteristics of LRM-ELNs

As illustrated in Figure 1B(a), increasing the PEG6000 concentration from 6% to 10% resulted in a rise in LRM-ELN yield from 2.62 g/kg to 3.50 g/kg. When the concentration further increased from 10% to 15%, the yield had no significant difference. As shown in Figure 1B(b), the PEG6000 concentration had no significant influence on zeta potential of LRM-ELNs. The particle size of the LRM-ELNs decreased dramatically (from 172.6 nm to 134.1 nm) as the PEG6000 concentration was raised from 8% to 10%. When the PEG6000 concentration further increased (from 10% to 15%), the particle size stayed around 140.0 nm (Figure 1B(c)). These results are consistent with Kalarikkal and coworkers’ work, which showed that the moderate increase in PEG6000 concentration leads to the higher yield and smaller size of ELN in plants [10]. This may be attributed to PEG6000 being a highly hydrophilic polymer that interacts with the water molecules surrounding ELNs, creating a hydrophobic microenvironment. At the appropriate PEG6000 concentration, the water solubility of the ELNs was reshaped, which resulted in more ELNs precipitating [35,36]. Considering the cost, extraction efficiency, and particle size of LRM-ELNs, 10% PEG6000 was considered as the optimal concentration for the LRM-ELN extraction.

#### 3.1.3. Effect of Relative Centrifugal Force on Yield and Characteristics of LRM-ELNs

The relative centrifugal force is an important factor related with the yield of ELNs. To investigate the influence of relative centrifugal force on yield and characteristics of LRM-ELNs, different relative centrifugal forces were employed. As relative centrifugal force increased from 2000× *g* to 8000× *g*, the yield of LRM-ELNs increased from 2.08 g/kg to 3.50 g/kg, while further increasing the relative centrifugal force from 8000× *g* to 10,000× *g* led to a plateau stage of yield of LRM-ELNs (Figure 1C(a)). When relative centrifugal force at the range from 2000× *g* to 10,000× *g*, the zeta potential of LRM-ELNs did not show a detectable change (Figure 1C(b)). When relative centrifugal force was increased from 2000× *g* to 6000× *g*, the particle size of sedimented LRM-ELNs decreased (from 169.77 nm to 131.33 nm) (Figure 1C(c)). However, further increasing the centrifugal force to 10,000× *g* did not alter the particle size (Figure 1C(c)). This is consistent with the study on the extraction of ELNs from ginger [10]. Therefore, considering both yield and particle size, the optimal relative centrifugal force was chosen as 8000× *g*.

#### 3.1.4. Effect of Incubation Time on Yield and Characteristics of LRM-ELNs

As shown in Figure 1D(a), the yield of the LRM-ELNs increased (from 1.75 g/kg to 3.89 g/kg) with the increase in incubation time (from 8 h to 20 h). When the incubation time was extended to 24 h, the yield showed a slight decrease (form 3.89 g/kg to 3.64 g/kg). As shown in Figure 1D(b), the incubation time showed no significant effect on zeta potential of LRM-ELNs. According to Figure 1D(c), the particle size decreased (from 184.3 nm to 135.3 nm) as incubation time increased from 8 to 20 h and then increased (from 135.3 nm to 199.2 nm) for further extended time. This may be due to prolonged incubation with PEG6000 leading to the aggregation of LRM-ELNs, where multiple LRM-ELN particles cluster together to form larger complexes [37]. Considering the yield and particle size of the LRM-ELNs, the optimal incubation time was chosen as 20 h.

### 3.2. Optimization of Parameters of LRM-ELN Extraction Using RSM

Considering that the LRM-ELN yield was influenced by the interactions of multiple factors, it is imperative to undertake further investigation and assessment of the individual factors and their interactions on the extraction efficiency. Based on previous single-factor experiments, three independent parameters, viz., PEG6000 concentration (A, 8–12%), relative centrifugal force (B, 6000–10,000× *g*), and incubation time (C, 16–24 h), were used as decision variables in this study and evaluated by various combinations. A total of 17 randomly arranged experiments and the corresponding responses (yield of LRM-ELNs) are summarized in Table S1. These results were input into the “Design Expert 13” software, a nonlinear quadratic mathematical model and point prediction tool, to assess the fitness of each model with ANOVA and F-test [38].

To ensure the applicability of the developed model at a 95% confidence level, the *p*-value of the model must be less than 0.05 [39]. According to Table 1, it can be observed that the *p*-value of the model is <0.0001, indicating a high level of confidence in the model for optimizing the LRM-ELN yield. In addition, the R^2^ value is 0.9856, which is close to the adjusted R^2^ (0.9671) and predicted R^2^ (0.9561) values, and indicates a high correlation between the model’s predicted outcomes and experimental results in this validation. These results demonstrated that the established model possesses good usability and accuracy [40]. Additionally, the lack of fit *p*-value is 0.9225 > 0.05, demonstrating that the lack of fit can be disregarded in influencing the results during the optimization testing process [41]. Furthermore, the coefficient of variation (C.V.) is less than 10%, indicating that the model exhibits good precision and reproducibility [42].

The impact of each factor on the model is considered highly significant (*p* ≤ 0.0001), and according to the F-values, the order of the influence of extraction conditions on LRM-ELN yield is as follows: A (concentration of PEG6000) > B (relative centrifugal force) > C (incubation time), indicating that the concentration of PEG6000 is the key factor affecting the yield. The interaction terms AB (*p*-value = 0.0033), AC (*p*-value = 0.1608), and BC (*p*-value = 0.0509) indicate that the AB interaction has a significant impact on LRM-ELN yield, while the AC and BC interactions do not significantly impact it. When the response values are regressed against the variable factors, the second-order regression equation for the composite index is obtained as follows:

$$\mathrm{Yield}=3.87+0.2475\;\times \;A+0.2013\;\times \;B+0.1588\;\times \;C+0.1250\;\times \;\mathrm{AB}+0.0450\;\times \;\mathrm{AC}+0.0675\;\times \;\mathrm{BC}\;-\;0.1498\;\times \;A^{2}\;-\;0.1423\;\times \;B^{2}\;-\;0.2422\;\times \;C^{2}$$

**Figure 2:**
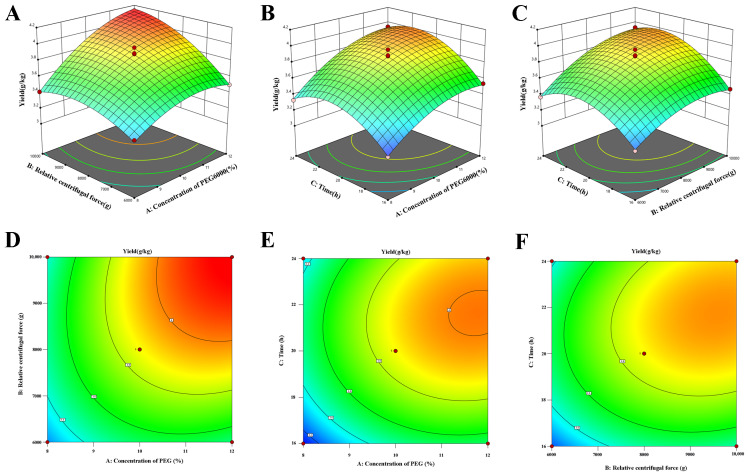
Three-dimensional (3D) response surface and the corresponding two-dimensional (2D) contour plots of the effect of the concentration of PEG6000, relative centrifugal force, and incubation time on the yield of LRM-ELNs: (**A**,**D**) concentration of PEG6000 and relative centrifugal force, (**B**,**E**) concentration of PEG6000 and time, (**C**,**F**) relative centrifugal force and time.

The surface response plots for the regression equation representing the yield of the LRM-ELNs are shown in Figure 2. The optimal extraction process has a PEG6000 concentration of 11.93%, centrifugal force of 9720× *g*, and an incubation time of 21.12 h. In this context, the predicted yield of the LRM-ELNs was 4.19 g/kg. To validate the suggested parameters, experiments were carried out in triplicate under optimized conditions, and the actual yield was 4.24 g/kg. The deviation between the actual measured value and the predicted value is less than 1.12%, indicating the reliability of the response surface optimization scheme.

### 3.3. The Characteristics of LRM-ELNs

**Figure 3:**
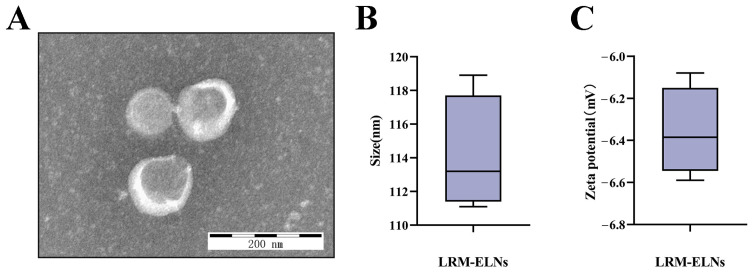
Characterization of LRM-ELNs. (**A**) LRM-ELNs were observed using TEM. (**B**) Particle size of the LRM-ELNs. (**C**) Zeta potential of the LRM-ELNs.

TEM was utilized to examine the morphology of LRM-ELNs isolated under optimized conditions. As depicted in Figure 3A, LRM-ELNs exhibited a spherical shape, representative of ELNs [40]. Analysis with a nanoparticle size analyzer revealed a mean particle size of 114.1 nm (Figure 3B). Additionally, the zeta potential of the LRM-ELNs was measured to be −6.36 mV (Figure 3C).

### 3.4. Effect of LRM-ELNs on Apoptosis in HT22 Cells Induced by Aβ

**Figure 4:**
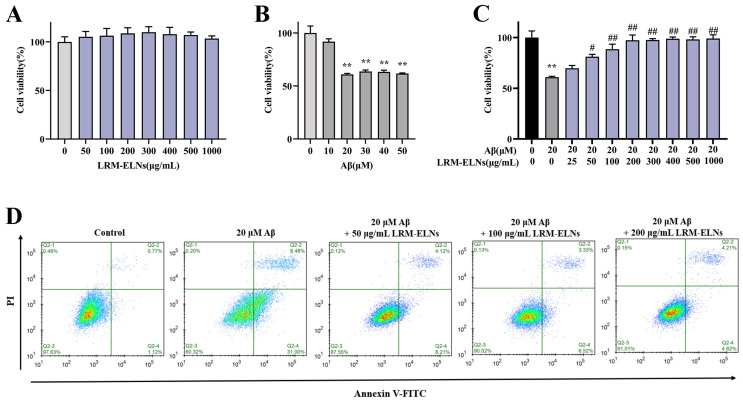
LRM-ELNs reduced Aβ-induced cytotoxicity and apoptosis in HT22 cells. The MTT assay was conducted to assess the impacts of (**A**) LRM-ELNs, (**B**) Aβ, and (**C**) LRM-ELNs/Aβ on the viability of HT22 cells. (**D**) Flow cytometry was conducted to evaluate the influence of LRM-ELNs on Aβ-induced apoptosis in HT22 cells. Data are expressed as mean ± SEM. ** *p* < 0.01 vs. the control group, # *p* < 0.05, and ## *p* < 0.01 vs. the Aβ group.

To assess the cytotoxicity of the LRM-ELNs on HT22 cells, the MTT assay was employed to determine cell viability. The LRM-ELNs, across a range of 0 to 1000 μg/mL, did not show significant cytotoxicity in HT22 cells (Figure 4A). Furthermore, as shown in Figure 4B, treatment with 20 μM Aβ reduced cell viability to 60.9%. To explore the defensive role of LRM-ELNs on HT22 cells treated with Aβ, the cells were exposed to a combination of Aβ and LRM-ELNs. As shown in Figure 4C, the viability of HT22 cells exposed to Aβ was enhanced from 60.9% to 97.3% with rising concentrations of LRM-ELNs ranging from 0 to 200 μg/mL. When the concentration of LRM-ELNs was raised to 300 μg/mL, cell viability remained constant and did not increase further. Therefore, LRM-ELN concentrations below 200 μg/mL were used for subsequent experiments.

The protective impact of the LRM-ELNs against Aβ-induced cell apoptosis was utilized using a flow cytometer. As demonstrated in Figure 4D, the addition of the LRM-ELNs decreased the apoptosis rate of Aβ-treated HT22 cells from 39.48% to 8.83%, which indicates that LRM-ELNs could inhibit Aβ-induced cell apoptosis in a dose-dependent manner.

### 3.5. Effects of LRM-ELNs on Mitochondrial Apoptosis in HT22 Cells Induced by Aβ

**Figure 5:**
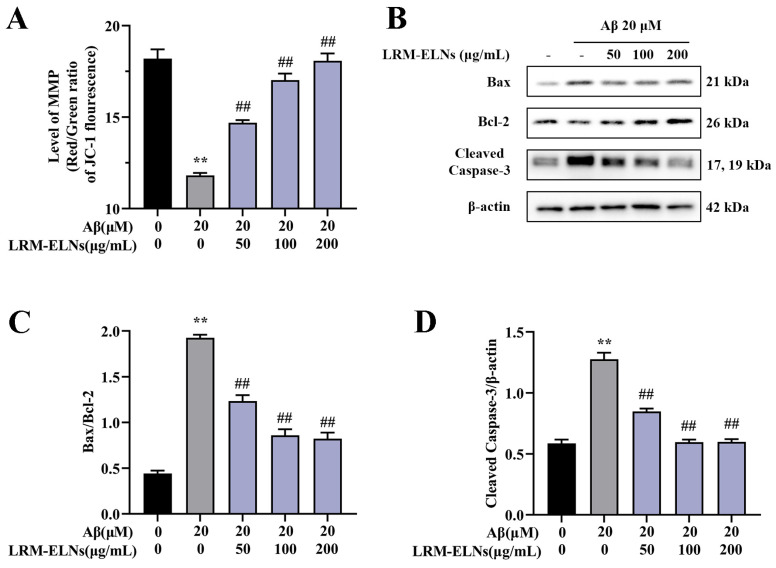
Effects of LRM-ELNs on the mitochondrial apoptosis pathway in HT22 cells induced by Aβ. (**A**) MMP in HT22 cells treated with Aβ, with or without different concentrations of LRM-ELNs. (**B**–**D**) The protein levels of Bax/Bcl2 and Cleaved Caspase-3 in HT22 cells were determined by WB. Data are expressed as mean ± SEM. ** *p* < 0.01 vs. the control group, ## *p* < 0.01 vs. the Aβ group.

Aβ has been shown to induce mitochondrial dysfunction, which in turn leads to neuronal apoptosis [43]. Studies have demonstrated that mitochondrial damage is characterized by a decrease in mitochondrial membrane potential (MMP) [44]. As shown in Figure 5, the incubation of HT22 cells with Aβ markedly decreased the MMP, increased the Bax/Bcl-2 ratio, and elevated the levels of Cleaved Caspase-3. However, when LRM-ELNs were introduced to the Aβ-treated cells, there was an increase in MMP, a decrease in the Bax/Bcl-2 ratio, and a reduction in Cleaved Caspase-3 expression. These findings suggest that LRM-ELNs confer protective effects against Aβ-induced damage in HT22 cells by inhibiting mitochondria-mediated apoptosis and promoting mitochondrial repair.

### 3.6. Effect of LRM-ELNs on the Accumulation of ROS and MDA and the Activities of Antioxidant Enzymes in Aβ-Treated HT22 Cells

**Figure 6:**
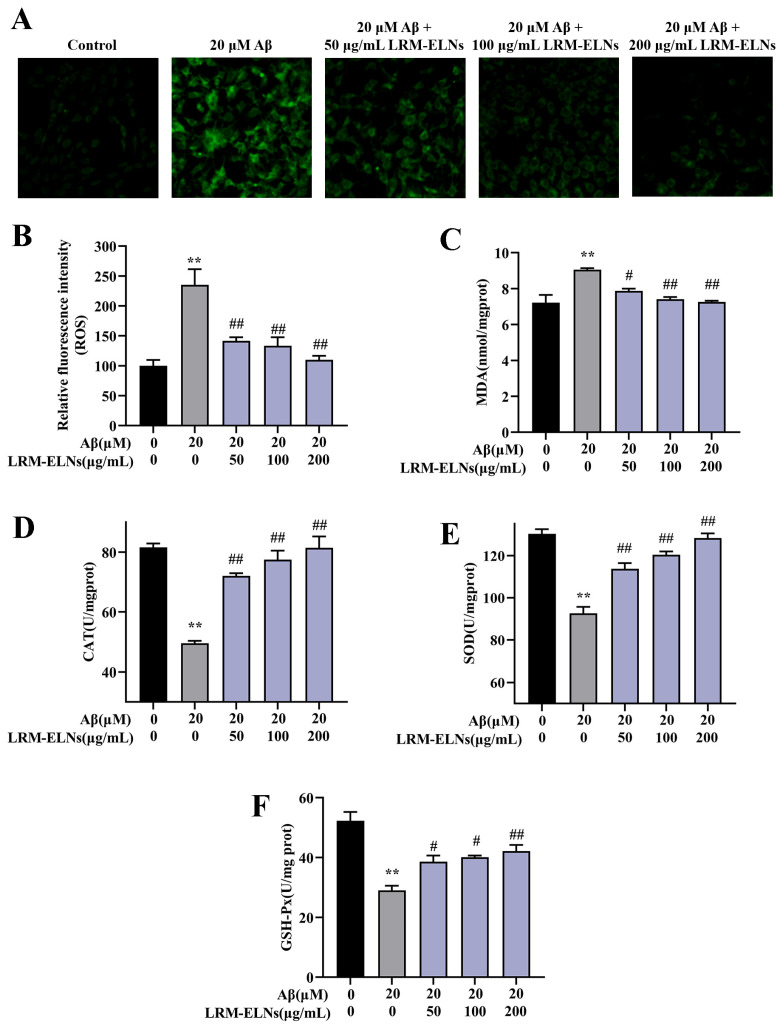
Effects of LRM-ELNs on antioxidant indices in HT22 cells. Levels of (**A**,**B**) ROS, (**C**) MDA, (**D**) CAT, (**E**) SOD, and (**F**) GSH-Px in HT22 cells treated with Aβ, with or without different concentrations of LRM-ELNs. Data are expressed as mean ± SEM. ** *p* < 0.01 vs. the control group, # *p* < 0.05, ## *p* < 0.01 vs. the Aβ group.

Aβ induces oxidative damage to neurons, affecting the levels of intracellular ROS and MDA, along with the activity of intracellular CAT, SOD, and GSH-Px [45,46]. As shown in Figure 6A,B, intracellular ROS levels increased with Aβ incubation, while the addition of LRM-ELNs reduced ROS levels. Additionally, LRM-ELNs reduced MDA levels induced by Aβ in HT22 cells (Figure 6C). Moreover, LRM-ELNs increased the activities of CAT, SOD, and GSH-Px in Aβ-treated HT22 cells (Figure 6D–F). These results indicate that LRM-ELNs can lower ROS levels, reduce lipid peroxidation, and enhance the activities of antioxidant enzymes CAT, SOD, and GSH-Px.

### 3.7. Effects of LRM-ELNs on the Nrf2/HO-1/NQO1 Signaling Pathway in in HT22 Cells Treated with Aβ

**Figure 7:**
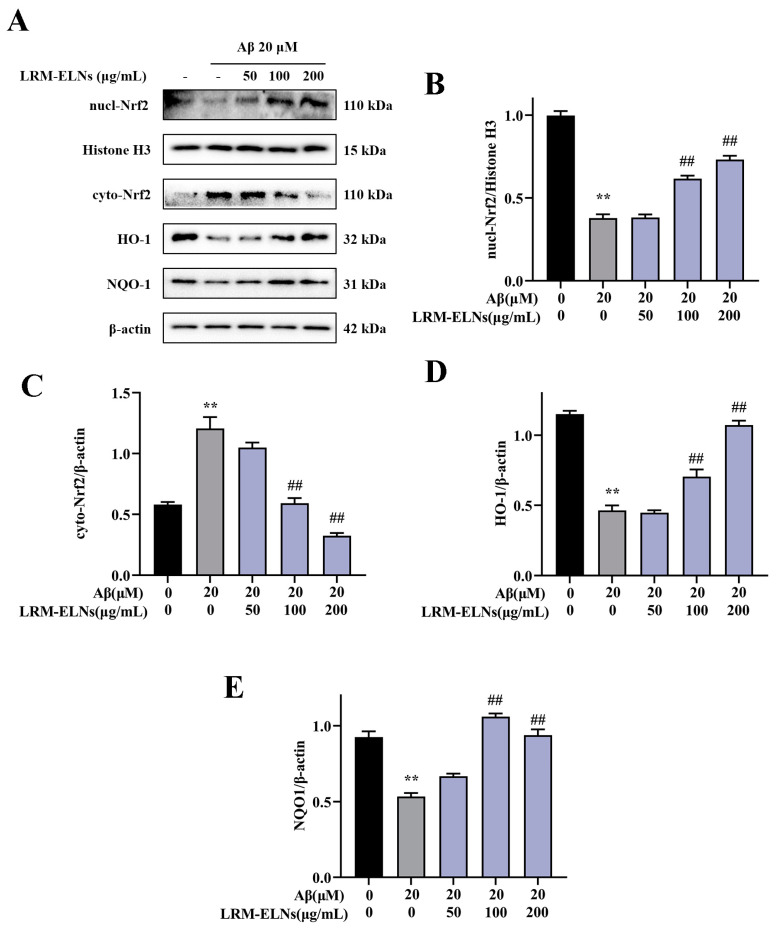
Effects of LRM-ELNs on the Nrf2/HO-1/NQO1 signaling pathway in HT22 cells induced by Aβ. (**A**–**E**) The protein levels of nucl-Nrf2, cyto-Nrf2, HO-1, and NQO1 in HT22 cells were determined by WB. Data are expressed as mean ± SEM. ** *p* < 0.01 vs. the control group, ## *p* < 0.01 vs. the Aβ group.

The Nrf2/HO-1/NQO1 signaling pathway is a vital antioxidant mechanism that regulates multiple processes to prevent cellular oxidative stress [47]. To assess the activation of the Nrf2/HO-1/NQO1 signaling pathway by LRM-ELNs in HT22 cells, protein expression levels were measured via WB analysis. As shown in Figure 7, Aβ treatment significantly increased cytoplasmic Nrf2 (cyto-Nrf2) expression in HT22 cells, whereas LRM-ELNs further decreased cyto-Nrf2 levels. Conversely, Aβ treatment reduced nuclear Nrf2 (nucl-Nrf2) levels, while LRM-ELNs increased nuclear Nrf2 levels, subsequently enhancing HO-1 and NQO1 expression.

## 4. Discussion

Plant-derived ELNs have demonstrated a range of biological activities [8,9]. These ELNs are rich in bioactive compounds and serve as effective carriers for proteins, lipids, DNA, and RNA [48]. A recent precipitation method utilizing PEG has been described for the targeted isolation of ELNs derived from ginger. This technique produces ginger-derived ELNs with similar yield and activity, providing a more economical and scalable method for producing plant-derived ELNs [10]. Several studies have utilized PEG to extract plant-derived ELNs, showing benefits like reducing mitochondrial oxidative stress in non-alcoholic fatty liver disease using blueberry ELNs and modulating liver dysfunction in high-fat diet mice with garlic ELNs [11,49]. These findings suggest that PEG-based separation is a promising method for extracting ELNs from plants. Therefore, this study comprehensively analyzed the effects of PEG molecular weights, concentrations, incubation times, and centrifugal forces on LRM-ELN yield. This approach aimed to optimize the extraction method for LRM-ELNs. The Box–Behnken design in RSM was used to optimize the extraction process by analyzing interactions among various factors. The results indicated that PEG6000 is the most suitable molecular weight for extracting LRM-ELNs. The optimal extraction process, with PEG6000 concentration at 11.93%, centrifugal force at 9720× *g*, and incubation time of 21.12 h, achieved a maximum yield of LRM-ELNs, with an actual yield of 4.24 g/kg under these conditions.

The pathology of AD shows a strong relationship between Aβ accumulation and the degree of dementia symptoms [19,20]. Mitochondria are essential for cellular respiration, metabolic process maintenance, and ion transport, significantly influencing the cell’s life cycle [50]. Research has shown that Aβ induces mitochondrial damage in neuronal cells, characterized by a decrease in mitochondrial membrane potential (MMP) [51]. This subsequently induces the expression of pro-apoptotic proteins like Bax, inhibits anti-apoptotic proteins like Bcl-2, and activates Cleaved Caspase-3, leading to apoptosis [46]. Our results showed that Aβ stimulation decreased MMP while increasing the Bax/Bcl-2 protein ratio and Cleaved Caspase-3 expression in HT22 cells. However, LRM-ELNs upregulated MMP, decreased the Bax/Bcl-2 ratio, and downregulated Cleaved Caspase-3 expression (Figure 5). Additionally, LRM-ELNs protected HT22 cells from Aβ-induced cytotoxicity (Figure 4C) and inhibited Aβ-induced apoptosis (Figure 4D). These findings suggest that LRM-ELNs protect HT22 cells from Aβ-induced apoptosis through the mitochondrial apoptotic pathway.

According to the amyloid cascade hypothesis, Aβ and its aggregates induce oxidative stress, resulting in neurodegeneration, neuronal dysfunction, and apoptosis [21,52,53]. Oxidative stress leads to excessive production of ROS, contributing to various pathological processes. For instance, lipid peroxidation can lead to the formation of harmful MDA, which may cross-link and damage cellular proteins and nucleic acids. Additionally, this process can impair the antioxidant defense system, affecting key enzymatic scavengers such as SOD, CAT, and GSH-Px. In this study, LRM-ELN treatment reduced the levels of oxidative products ROS and MDA in HT22 cells exposed to Aβ (Figure 6A–C). LRM-ELN treatment enhanced the activities of antioxidant enzymes SOD, CAT, and GSH-Px (Figure 6E,F). Nrf2, a crucial redox-sensitive transcription factor, is essential for maintaining cellular redox balance and mitigating oxidative damage [54]. The activation of Nrf2 signaling closely regulates the expression of antioxidant enzymes like SOD, CAT, and GSH-Px [54]. HO-1 by-products have strong ROS scavenging capabilities, while NQO1 by-products protect against DNA damage induced by environmental stressors [55,56]. Our research found that Aβ stimulation decreased nuclear Nrf2 expression and the levels of HO-1 and NQO1 in HT22 cells. LRM-ELNs significantly increased nuclear Nrf2 expression and upregulated NQO1 and HO-1 in a dose-dependent manner (Figure 7), offering protection against Aβ-induced oxidative stress in HT22 cells.

Plant-derived ELNs contain lipids, proteins, DNA, and RNA (such as small RNA (sRNA) and microRNA (miRNA)), and play a crucial role in intercellular communication [57]. MiRNA is a non-coding RNA that silences target mRNA by binding to the 3′-untranslated region (UTR) or the open reading frame (ORF) [58]. Plant-derived ELNs serve as excellent carriers of miRNA, leading to increased attention on the role of miRNA within ELNs [29]. Our previous research found that LRM-ELNs are composed of lipids, proteins, and RNA and that LRM-ELNs have an inhibitory effect on Aβ-induced apoptosis in PC12 cells, with miRNAs playing a key role [28]. Additionally, miR-7972 derived from fresh Rehmanniae Radix ELNs has been shown to inhibit LPS-induced ROS production in RAW264.7 cells [59]. Given this, we suppose that LRM-ELNs may exert inhibitory effects on Aβ-induced apoptosis and oxidative stress in HT22 cells through miRNA delivery, and we will further verify and investigate this mechanism in future studies.

## 5. Conclusions

In summary, the optimal conditions for extracting LRM-ELNs using the PEG method were a PEG6000 concentration of 11.93%, a centrifugal force of 9720× *g*, and an incubation time of 21.12 h, resulting in an LRM-ELN yield of 4.24 g/kg. Further investigation revealed that LRM-ELNs reversed Aβ-induced reductions in MMP and decreased the Bax/Bcl-2 ratio and Cleaved Caspase-3 expression, thereby reducing apoptosis in HT22 cells. Additionally, the LRM-ELNs activated the Nrf2/HO-1/NQO1 signaling pathway, increasing the expression of SOD, CAT, and GSH-Px, and reducing ROS and MDA levels, thereby mitigating Aβ-induced oxidative stress in HT22 cells. These findings suggest that LRM-ELNs could be a promising candidate for AD treatment.

## Figures and Tables

**Table 1:** ANOVA of the response surface model and predicted result for the response of the analyte.

Source	Sum of Squares	df	Mean Square	F-Value	*p*-Value	
Model	1.58	9	0.1752	53.20	<0.0001	significant
A—Concentration of PEG	0.4901	1	0.4901	148.79	<0.0001	
B—Relative centrifugal force	0.3240	1	0.3240	98.38	<0.0001	
C—Time	0.2016	1	0.2016	61.21	0.0001	
AB	0.0625	1	0.0625	18.98	0.0033	
AC	0.0081	1	0.0081	2.46	0.1608	
BC	0.0182	1	0.0182	5.53	0.0509	
A^2^	0.0944	1	0.0944	28.67	0.0011	
B^2^	0.0852	1	0.0852	25.87	0.0014	
C^2^	0.2471	1	0.2471	75.02	<0.0001	
Residual	0.0231	7	0.0033			
Lack of Fit	0.0024	3	0.0008	0.1531	0.9225	not significant
Pure Error	0.0207	4	0.0052			
Cor Total	1.60	16				

R^2^ = 0.9856, Adjusted R^2^ = 0.9671, Predicted R^2^ = 0.9561, C.V. % = 1.59. Predicted value = 4.19 g/kg, Actual value = 4.24 g/kg, Error in relation to predicted value (%) = 1.12.

## Data Availability

The original contributions presented in the study are included in the article/Supplementary Materials, further inquiries can be directed to the corresponding author.

## Supplementary Materials

The following supporting information can be downloaded at: https://www.mdpi.com/article/10.3390/foods13203328/s1, Figure S1: Experimental flowchart illustrating the procedure for isolating LRM-ELNs. Table S1: The parameters and corresponding results in the Box–Behnken design (BBD) experiment.
