# Supplementary material for: Response Surface Methodology Optimization of Exosome-like Nanovesicles Extraction from Lycium ruthenicum Murray and Their Inhibitory Effects on Aβ-Induced Apoptosis and Oxidative Stress in HT22 Cells
Source: Foods. 2024 Oct 20;13(20):3328. doi: 10.3390/foods13203328 (PMC11507227; doi:10.3390/foods13203328)
Supplement: Supplementary file 1 [file foods-13-03328-s001.zip › foods-3240382-supplementary.pdf]

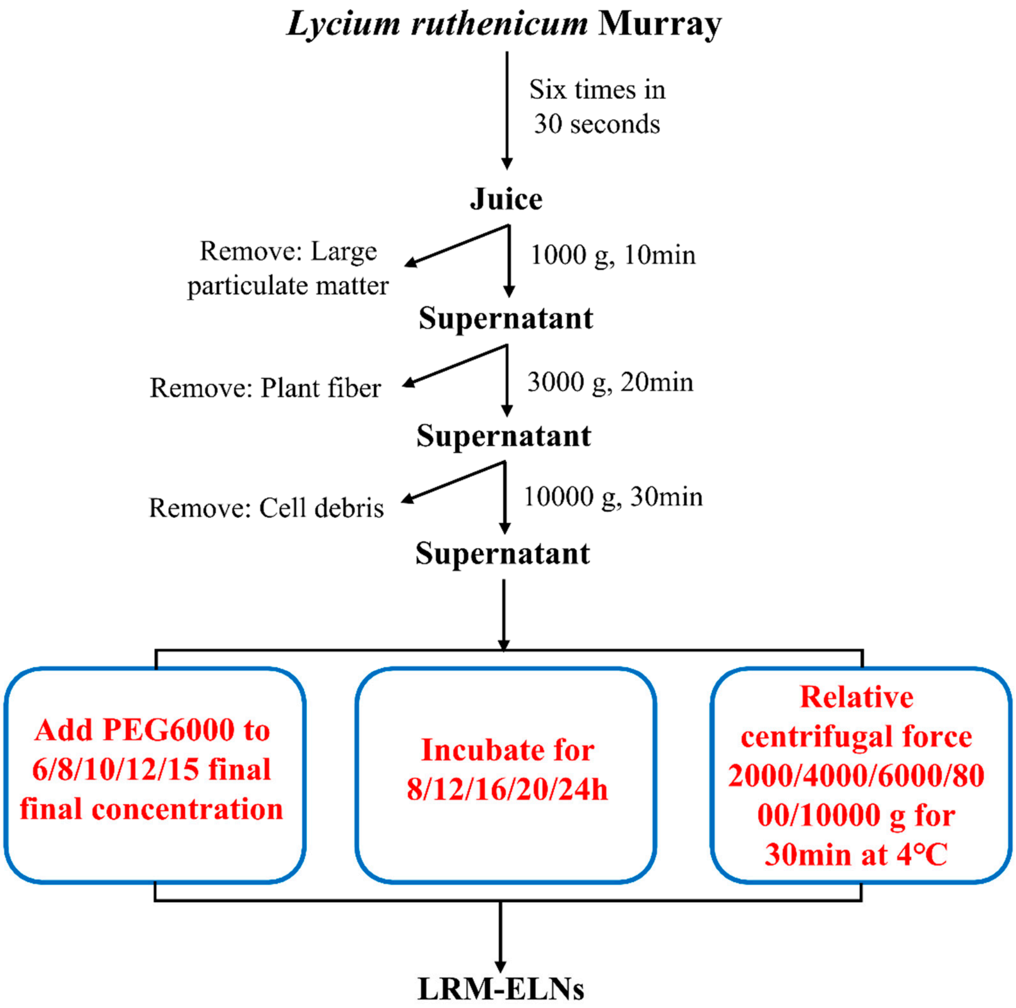

Figure S1 Experimental flowchart illustrating the procedure for isolating LRM-ELNs.

Table S1 The parameters and corresponding results in the Box-Behnken Design (BBD) experiment.

Std	Run	Concentration of PEG6000 (%)	Relative centrifugal force (g)	Time (h)	Yield (%)
8	1	12	8000	24	3.94
12	2	10	10000	24	3.93
17	3	10	8000	20	3.87
16	4	10	8000	20	3.89
15	5	10	8000	20	3.88
1	6	8	6000	20	3.28
13	7	10	8000	20	3.96
6	8	12	8000	16	3.54
9	9	10	6000	16	3.18
14	10	10	8000	20	3.76
2	11	12	6000	20	3.50
11	12	10	6000	24	3.37
3	13	8	10000	20	3.41
5	14	8	8000	16	3.11
7	15	8	8000	24	3.33
10	16	10	10000	16	3.47
4	17	12	10000	20	4.13
